# Computation of molecular description of supramolecular Fuchsine model useful in medical data

**DOI:** 10.1038/s41598-024-60284-3

**Published:** 2024-05-13

**Authors:** Zunaira Kosar, Shahid Zaman, Asad Ullah, Muhammad Kamran Siddiqui, Melaku Berhe Belay

**Affiliations:** 1 Department of Mathematics, University of Sialkot, Sialkot, 51310 Pakistan; 2 Department of Mathematical Sciences, Karakoram International University Gilgit-Baltistan, Gilgit, 15100 Pakistan; 3 Department of Mathematics, Comsats University Islamabad, Lahore Campus, Lahore, Pakistan; 4 Nanotechnology Center of Excellence, Addis Ababa Science and Technology University, Mathematics, Physics and Statistics Division, Addis Ababa Science and Technology University, P. O. Box 16417, Addis Ababa, Ethiopia

**Keywords:** Molecular descriptor, Fuchsine $C_20H_19N_3HCl$, Topological descriptors, Applied mathematics, Cheminformatics

## Abstract

Supramolecular chemistry is a fascinating field that explores the interactions between molecules to create higher-order structures. In the case of the supramolecular chain of Fuchsine acid, which is a type of dye molecule, several chemical applications are possible. Fuchsine acid helps to make better medicine carriers that deliver drugs where they’re needed in the body, making treatments more effective and reducing side effects. It also helps create smart materials like sensors and self-fixing plastics, which are useful in electronics, keeping our environment clean, and making new materials. In sensing and detection, the supramolecular chain of Fuchsine acid utilizes as a sensor or detector for specific analyzes. In drug delivery, the supramolecular chains of Fuchsine acid incorporated into drug delivery systems. In recent years, a common method is linking a graph to a chemical structure and using topological descriptors to study it. This technique is becoming increasingly important over time. Topological descriptors gives very useful information while studying the topology of chemical graph. In this paper, we have computed the 3*D* structure of supramolecular graph of Fuchsine acid. We have computed an explicit expressions of ABC index, GA index, General Randi$\acute{c}$ index, first and second Zagreb index, hyper Zagreb index, H-index and F-index of supramolecular structure of Fushine acid.

## Introduction

A graph associated to structural formulas which include covalent bonded compounds or molecules, hence they are called molecular graphs. In the field of theoretical chemistry a large number of topological descriptors are used by pharmaceutical researchers. To study a chemical structure one can use graph theory, where atoms are considered as vertices of graph and molecular bond represents the edges. Topological descriptors are numbers linked to the structure, helping predict its properties like how it behaves chemically or physically. This numeric carry useful and important information about chemical structure and is usually called as topological index^1^. A topological index is a graph invariant under graph automorphism. Topological indices are numerical descriptors used to characterize the structure of graphs^2^. They play a crucial role in correlating the topology of molecular graphs with various physio-chemical properties such as boiling point, viscosity, and radius of gyrations^3,4^. Additionally, these indices are useful in predicting bioactivity of molecules^5–7^. They find applications in diverse fields such as drug design, environmental chemistry, and material science^8–14^.

A branch of chemistry named Supramolecular chemistry deals with chemical systems which are the composition of molecules. In supramolecular chemistry without outside guidance and providing the suitable environment new chemical systems can be constructed, this concept is called Molecular self-assembly. The molecules are directed to assemble through non-covalent interactions. Fuchsine $C_20H_19N_3HCl$ is a magenta dye. Fuchsine is used for dyeing clothes, staining bacteria, and sometimes as a disinfectant because of its important properties. In the study of biological tissues stains are used to increase contrast in sample, this technique is called staining. Dyes and stains are widely utilized in various applications, with one of the most common uses being in the microscopic study of biological tissues.

In the medical fields of histopathology and cytopathology, dyes and stains play a crucial role in focusing at the microscopic level for disease detection and analysis of tissue samples. Biological tissues also can be defined by stains, for example it is used for emphasizing muscle fibers or connective tissue, also used to categorize different blood cells. A staining method called Gram’s method, is used to sort bacterial species into two huge groups Gram-positive bacteria and Gram-negative bacteria. The Gram staining method often uses a dye called Fuchsine in microbiology. Fuchsine is a cog in the Schiff test, which is developed by Hugo Schiff and is an early organic chemistry named reaction. It is a comparatively common chemical test for finding of many organic aldehydes.

The graphs we take into consideration here are all connected, simple, and finite. The vertex set and edge set of the graph *G* are denoted by $V_G$ and ${E_G}$ respectively. If there is an edge connecting two vertices, they are considered to be adjacent. We use the notation $xy\in E_G$ if there is an edge between *x* and *y*. For a vertex $x\in V_G$, we denote its degree by $\mathfrak {n}(x)$ and is defined as the number of edges incident to that vertex. For basic definitions and related graph theory notions, we refer the readers to the book by^15^.

In the field of mathematical chemistry, graphs can be associated with various mathematical representations, including polynomials, numeric values, or matrices. These representations are often referred to as molecular descriptors, which play a crucial role in quantitative structure-property relationship (QSPR) and quantitative structure-activity relationship (QSAR) investigations. Notably, topological indices are essential examples of such molecular descriptors. Nowadays, there are various topological indices are used get important information about chemical structures and undirected networks. Topological descriptors can be classified into three main types: distance-based, degree-based, and counting-related. Among these, degree-based topological descriptors have received significant attention and find applications in QSPR analysis. The first degree-based topological index was introduced by Randi$\acute{c}$^16^ in 1975 with the name branching index. This index is defined as

$$\begin{aligned} R_{-\frac{1}{2}}(G)=\sum _{xy \in E_G}\frac{1}{(\mathfrak {n}((x)\mathfrak {n}(y))^{\frac{1}{2}}}. \end{aligned}$$

Randi$\acute{c}$ introduced an index suitable for quantifying the extent of branching in the carbon-atom skeleton of saturated hydrocarbons. He observed a strong correlation between the Randi$\acute{c}$ index and various physical/chemical properties of Alkanes, such as boiling points, enthalpies of formation, and surface areas. Later in 1988, Bollob’as and Erdős generalized this concept by replacing the factor $\frac{-1}{2}$ in the Randi$\acute{c}$ index formula with a real number $\lambda$. The formula for general Randi$\acute{c}$ index is given below:

$$\begin{aligned} R_{\lambda }(G)=\sum _{xy \in E_G}(\mathfrak {n}(x)\mathfrak {n}(y))^\lambda \end{aligned}$$

For further details and important results about Randi$\acute{c}$ index see^17–19^.

Estrada et al. introduced a specific index known as the Atom Bond Connectivity index of a graph G, denoted as ABC(G). This index is defined as follows^3^:

$$\begin{aligned} ABC(G)=\sum _{xy \in E_G}\left( \frac{\mathfrak {n}(x)+\mathfrak {n}(y)-2}{\mathfrak {n}(x)\mathfrak {n}(y)}\right) ^{\frac{1}{2}}. \end{aligned}$$

Estrada proved that ABC index show a good model for the stability of linear and branched alkanes^3^.

The Geometric Arithmetic index, denoted as GA, was introduced by Vuki$\check{c}$evi$\acute{c}$ et al.^20^. For a given graph G, the Geometric Arithmetic index (GA) is formulated as follows:

$$\begin{aligned} GA(G)=\sum _{xy \in E_G}\frac{2(\mathfrak {n}(x)\mathfrak {n}(y))^{\frac{1}{2}}}{\mathfrak {n}(x)+\mathfrak {n}(y)}. \end{aligned}$$

In 1972 the first and second Zagreb indices were introduced^21,22^. These indices are denoted and defined as:

$$\begin{aligned} M_1(G)=\sum _{xy \in E_G} (\mathfrak {n}(x)+\mathfrak {n}(y)).\\ M_2(G)=\sum _{xy \in E_G} (\mathfrak {n}(x)\mathfrak {n}(y)). \end{aligned}$$

These topological indices were firstly applied to branching problem in early seventies^23^. Different researchers used these topolgical indices in their QSPR, QSAR studies^1,24,25^.

In 2013 Shirdel et al.^26^ prposed the hyper-Zagreb index as:

$$\begin{aligned} HM(G)=\sum _{xy \in E_G} (\mathfrak {n}(x)+\mathfrak {n}(y))^2. \end{aligned}$$

In 2012 the harmonic index was introduced by Zhong et al.^4^ as:

$$\begin{aligned} H(G)=\sum _{xy \in E_G} \frac{2}{\mathfrak {n}(x)+\mathfrak {n}(y)}. \end{aligned}$$

Furtula and Gutman^27^ in 2015 introduced a new index named the forgotten index, denoted by *F*(*G*) and formulated as:

$$\begin{aligned} F(G)=\sum _{x_ix_j \in E_G} (\mathfrak {n}(x_i)^2+\mathfrak {n}(x_j)^2). \end{aligned}$$

In this work we consider the 3D structure of supramolecular Fuchsine $C_20H_19N_3HCl$. We made the sheet of supra molecular Fushine by attaching its $m\times n$ units. We have computed an exact formulas for the Atom bond connectivity index , Geometric Arithmetic index , General Randi$\acute{c}$ index and different variants of Zagreb indices of $C_20H_19N_3HCl$ sheet.

## Main results

**Figure 1:**
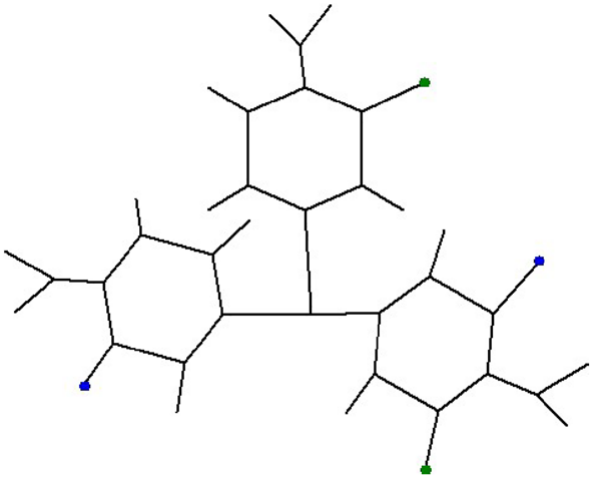
2*D* structure of *F*[1, 1].

**Figure 2:**
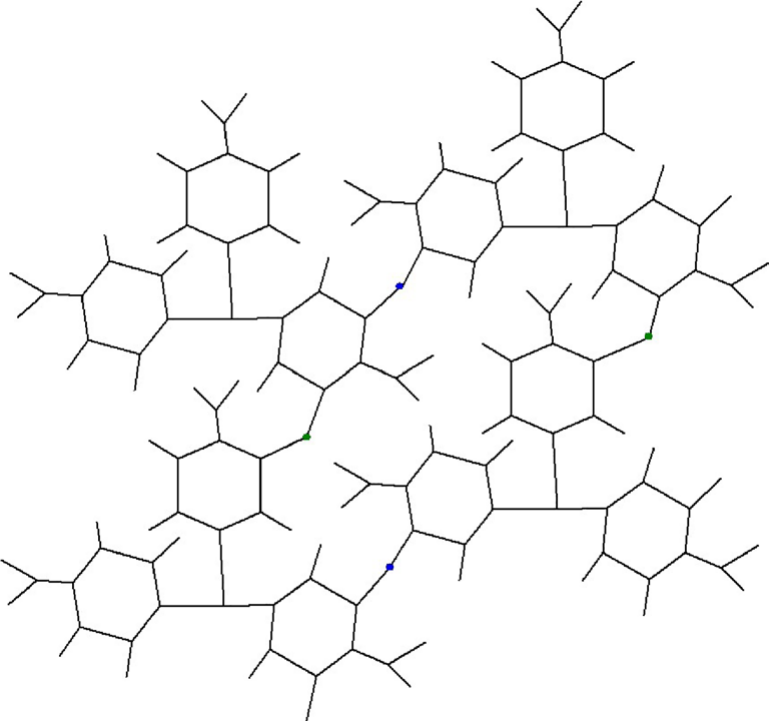
2*D* structure of *F*[2, 2].

We use the notation *F*[*m*, *n*] to denote the supramolcular structure of Fushine sheet having $m\times n$ units of Fushine. The single unit of Fushine is depicted in Fig. 1. The supramolecular sheet of Fushine *F*[*m*, *n*] is obtained by making a chain of *m* units of Fushine by connecting the molecules of Fushine with blue color vertex shown in the figure, as common vertex, and then connecting a *n* chains of *m* units of Fushine chain with green vertices. The molecular structure of *F*[2, 2] is shown in Fig. 2. It is easy to observe that *F*[*m*, *n*] has $38mn+m+n$ vertices and 42*mn* edges.

To compute the topological indices defined above, we need to find the partition of edges of *F*[*m*, *n*] based on the degree of end vertices. There are three types of edges of *F*[*m*, *n*]. The first edge partition has 24*mn* edges *xy* with ${\mathfrak {n}}(x)=3$ and $\mathfrak {n}(y)=3$. The second edge partition has $14mn+2(m+n)$ edges *xy* with ${\mathfrak {n}}(x)=3$ and $\mathfrak {n}(y)=1$. The third edge partition contains $n(2m-2)+2m(n-1)$ edges *xy*, where ${\mathfrak {n}}(x)=2$ and $\mathfrak {n}(y)=3$. The Table 1 shows the edge partition of *F*[*m*, *n*] with $m,n\ge 1$.

**Table 1:** Edge partition of *F*[*m*, *n*] based on degrees of end vertices of each edge.

$(\mathfrak {n}(x),\ \mathfrak {n}(y))$	Frequency
$(3,\ 3)$	24*mn*
$(3,\ 1)$	$14mn+2(m+n)$
$(2,\ 3)$	$n(2m-2)+2m(n-1)$

In the first theorem, we calculate the Atom Bond Connectivity index of *F*[*m*, *n*].

### Theorem 2.1

Let $m,n\ge 1$, then the value of Atom Bond Connectivity index of *F*[*m*, *n*] is equal to

$$\begin{aligned} ABC(F[m,n])= & {} mn \left( 16+14\frac{\sqrt{2}}{3}+2\sqrt{2} \right) +(m+n) \left( 2\frac{\sqrt{2}}{3}-\sqrt{2}\right) . \end{aligned}$$

### Proof

By using the edge partition of *F*[*m*, *n*] based on the degree of end vertices of each edge given in Table 1, the Atom Bond Connectivity index can be computed as:

$$\begin{aligned} ABC(F[m,n])= & {} \sum _{xy \in E_G}\left( \frac{\mathfrak {n}(x)+\mathfrak {n}(y)-2}{\mathfrak {n}(x)\mathfrak {n}(y)}\right) ^{\frac{1}{2}}\\ ABC(F[m,n])= & {} (24mn)\sqrt{\frac{3+3-2}{3\times 3}}+(14mn+2(m+n))\sqrt{\frac{3+1-2}{3\times 1}}+(n(2m-2)+2m(n-1))\sqrt{\frac{2+3-2}{3\times 2}}. \end{aligned}$$

After some easy calculations, above expression get the following form:

$$\begin{aligned} ABC(F[m,n])= & {} mn \left( 16+14\frac{\sqrt{2}}{3}+2\sqrt{2} \right) +(m+n) \left( 2\frac{\sqrt{2}}{3}-\sqrt{2}\right) . \end{aligned}$$

$$\square$$

In the next theorem, we calculate the general Randi$\acute{c}$ index ($R_{\lambda }(G)$) of supramolecular graph of Fuchsine *F*[*m*, *n*].

### Theorem 2.2

The general Randi$\acute{c}$ index of graph *F*[*m*, *n*] , with $m,n\ge 1$, is equal to

$$\begin{aligned} R_{\lambda } (F[m,n]) = {\left\{ \begin{array}{ll} 282mn-6m-6n, &{} \text {if} \,\,\, \lambda =1, \\ \frac{24mn+m+n}{3}, &{} \text {if} \, \, \, \lambda =-1, \\ mn(72+14\sqrt{3}+4\sqrt{6})+(m+n)(2\sqrt{3}-2\sqrt{6}) &{} \text {if} \, \, \,\lambda =\frac{1}{2}, \\ mn(8+\frac{14+2\sqrt{2}}{\sqrt{3}})+(m+n)(2-\sqrt{2}) &{} \text {if} \, \,\, \lambda =-\frac{1}{2}. \end{array}\right. } \end{aligned}$$

### Proof

One can prove the above result by using edge partition given in Table 1 in the definition of General Randi$\acute{c}$ index. When $\lambda =1$.

$$\begin{aligned} R_{1}(F[m,n])= & {} \sum _{xy \in E_G}(\mathfrak {n}(x) \times \mathfrak {n}(y))\\ R_{1}(F[m,n])= & {} (24mn)(3\times 3)+(14mn+2(m+n))(3\times 1)+(n(2m-2)+2m(v-1))(3\times 2)\\ R_{1}(F[m,n])= & {} 282mn-6m-6n. \end{aligned}$$

The subsequent form for $\lambda =-1$ is,

$$\begin{aligned} R_{-1}(F[m,n])= & {} \sum _{xy \in E_G}\frac{1}{(\mathfrak {n}(x) \times \mathfrak {n}(y))}\\ R_{-1}(F[m,n])= & {} (24mn) \left( \frac{1}{3\times 3} \right) +(14mn+2(m+n)) \left( \frac{1}{3\times 1}\right) +(n(2m-2)+2m(n-1)) \left( \frac{1}{3\times 2}\right) \\ R_{-1}(F[m,n])= & {} \frac{24mn+m+n}{3}. \end{aligned}$$

For $\lambda =\frac{1}{2}$, the formula of Randi$\acute{c}$ index takes the subsequent form.

$$\begin{aligned} R_{\frac{1}{2}}(F[m,n])= & {} \sum _{xy \in E_G}\sqrt{(\mathfrak {n}(x) \times \mathfrak {n}(y))}\\ R_{\frac{1}{2}}(F[m,n])= & {} (24mn)(\sqrt{3\times 3})+(14mn+2(m+n))(\sqrt{3\times 1})+(n(2m-2)+2m(n-1))(\sqrt{3\times 2})\\ R_{\frac{1}{2}}(F[m,n])= & {} mn(72+14\sqrt{3}+4\sqrt{6})+(m+n)(2\sqrt{3}-2\sqrt{6}). \end{aligned}$$

The subsequent form for $\lambda =\frac{-1}{2}$ is,

$$\begin{aligned} R_{-\frac{1}{2}}(F[m,n])= & {} \sum _{xy \in E_G}\frac{1}{\sqrt{(\mathfrak {n}(x) \times \mathfrak {n}(y))}}\\ R_{-\frac{1}{2}}(F[m,n])= & {} (24mn)\left( \frac{1}{\sqrt{3\times 3}}\right) +(14mn+2(m+n))\left( \frac{1}{\sqrt{3\times 1}}\right) +(n(2m-2)+2m(n-1))\left( \frac{1}{\sqrt{3\times 2}}\right) \\ R_{-\frac{1}{2}}(F[m,n])= & {} mn \left( 8+\frac{14+2\sqrt{2}}{\sqrt{3}}\right) +(m+n)(2-\sqrt{2}). \end{aligned}$$

$$\square$$

Geometric arithmetic index *GA* of *F*[*m*, *n*] is calculated in the next theorem.

### Theorem 2.3

Let $m,n\ge 1$, then the geometric arithmetic index of *F*[*m*, *n*] is equal to

$$\begin{aligned} GA(F[m,n])= & {} mn \left( 24+14\frac{\sqrt{3}}{2}+8\frac{\sqrt{6}}{5} \right) +(m+n) \left( \sqrt{3}-4\frac{\sqrt{6}}{5}\right) . \end{aligned}$$

### Proof

Using the edge partition given in Table 1, the geometric arithmetic index is calculated as below:

$$\begin{aligned} GA(F[m,n])= & {} \sum _{xy \in E_G}\frac{2\sqrt{\mathfrak {n}(x)\mathfrak {n}(y)}}{\mathfrak {n}(x)+\mathfrak {n}(y)}\\ GA(F[m,n])= & {} (24mn)\frac{2\sqrt{9}}{3+3}+(14mn+2(m+n))\frac{2\sqrt{3}}{3+1}+(n(2m-2)+2m(n-1))\frac{2\sqrt{3}}{2+3}\\ GA(F[m,n])= & {} mn \left( 24+14\frac{\sqrt{3}}{2}+8\frac{\sqrt{6}}{5} \right) +(m+n) \left( \sqrt{3}-4\frac{\sqrt{6}}{5} \right) . \end{aligned}$$

$$\square$$

In the next theorem, we calculate the first and second Zagreb indices of *F*[*m*, *n*].

### Theorem 2.4

The values of first and second Zagreb indices of *F*[*m*, *n*], with $m,n\ge 1$, are equal to

$$\begin{aligned} M_1(F[m,n])= & {} 220mn-2m-2n\\ M_2(F[m,n])= & {} 282mn-6m-6n. \end{aligned}$$

### Proof

Using the values from Table 1, the value of first Zagreb index of *F*[*m*, *n*] can be computed as below:

$$\begin{aligned} M_1(F[m,n])= & {} \sum _{xy \in E_G} (\mathfrak {n}(x)+\mathfrak {n}(y)).\\ M_1(F[m,n])= & {} (24mn)(3+3)+(14mn+2(m+n))(3+1)+(n(2m-2)+2m(n-1))(2+3))\\ M_1(F[m,n])= & {} 220mn-2m-2n. \end{aligned}$$

The second Zagreb index is calculated below:

$$\begin{aligned} M_2(F[m,n])= & {} \sum _{xy \in E_G} (\mathfrak {n}(x)\mathfrak {n}(y))=R_{1}(G)\\ M_2(F[m,n])= & {} (24mn)(3\times 3)+(14mn+2(m+n))(3\times 1)+(n(2m-2)+2m(n-1))(2\times 3))\\ M_2(F[m,n])= & {} 282mn-6m-6n. \end{aligned}$$

$$\square$$

### Theorem 2.5

Let $m,n\ge 1$, then the hyper-Zagreb index of *F*[*m*, *n*] is equal to

$$\begin{aligned} HM(F[m,n])= & {} 118mn-18m-18n. \end{aligned}$$

### Proof

Using Table 1 the hyper-Zagreb index of *F*[*m*, *n*] can be computed as below:

$$\begin{aligned} HM(F[m,n])= & {} \sum _{xy \in E_G} (\mathfrak {n}(x)+\mathfrak {n}(y))^2.\\ HM(F[m,n])= & {} (24mn)(3+3)^2+(14mn+2(m+n))(3+1)^2+(n(2m-2)+2m(n-1))(2+3)^2\\ HM(F[m,n])= & {} 1188mn-18m-18n. \end{aligned}$$

$$\square$$

### Theorem 2.6

Let $m,n\ge 1$, then the harmonic index of *F*[*m*, *n*] is equal to

$$\begin{aligned} H(F[m,n])= & {} \frac{83}{5}mn+\frac{1}{5}(m+n). \end{aligned}$$

### Proof

Using Table 1, the harmonic index of *F*[*m*, *n*] can be computed as below:

$$\begin{aligned} H(F[m,n])= & {} \sum _{xy \in E_G} \frac{2}{\mathfrak {n}(x)+\mathfrak {n}(y)}.\\ H(F[m,n])= & {} (24mn) \left( \frac{2}{3+3}\right) +(14mn+2(m+n)) \left( \frac{2}{3+1}\right) +(n(2m-2)+2m(n-1)) \left( \frac{2}{2+3}\right) \\ H(F[m,n])= & {} \frac{83}{5}mn+\frac{1}{5}(m+n). \end{aligned}$$

$$\square$$

### Theorem 2.7

Let $m,n\ge 1$, then the forgotten index of *F*[*m*, *n*] is equal to

$$\begin{aligned} F(F[m,n])= & {} 624mn-6m-6n. \end{aligned}$$

### Proof

Let *F*[*m*, *n*] be the given graph. Using Table 1 the forgotten index of *F*[*m*, *n*] can be computed as below:

$$\begin{aligned} F({F[m,n]})= & {} \sum _{xy \in E_G} (\mathfrak {n}(x)^2+\mathfrak {n}(y)^2).\\ F(F[m,n])= & {} (24mn)(9+9)+(14mn+2(m+n))(9+1)+(n(2m-2)+2m(n-1))(9+4)\\ F(F[m,n])= & {} 624mn-6m-6n. \end{aligned}$$

$$\square$$

## Discussion

The importance of topological descriptors is due to the fact that they are usefull in QSPR/QSAR studies. In this work, we have computed the values of different degree based topological descritpors of supramolecular structure of Fushine. The values of these topological descriptors for different values of *m* and *n* are depicted in Table 2. Observe that the value of each index increses with the increase in the value of *m* and *n*. The plot of these indices help us to compare these indices. The Randi$\acute{c}$ index $R_{\frac{-1}{2}}$ has the maximum value among all these indices and the Randi$\acute{c}$ index $R_{-1}$ has the minimum value.

**Table 2:** Comparison of the topological indices.

*G*	$M_1(G)$	$M_2(G)$	*HM*(*G*)	*F*(*G*)	*ABC*(*G*)	*GA*(*G*)	$R_{-1}(G)$	$R_{1/2}(G)$	$R_{-1/2}(G)$	$R_1(G)$
*F*[1, 1]	216	270	1152	612	25	40	7	103	19	270
*F*[2, 2]	874	1104	4680	2472	101	159	33	419	72	1104
*F*[3, 3]	1968	2502	10,584	5580	227	359	74	946	162	2502
*F*[4, 4]	3504	4464	18,864	9936	405	625	131	1713	286	4464
*F*[5, 5]	5480	6990	29,520	15,540	635	999	203	2637	446	6990
*F*[6, 6]	7896	10,080	42,552	22,392	915	1439	340	3800	642	10,080
*F*[7, 7]	10,752	13,734	57,960	30,492	1246	1901	397	5176	873	13,734
*F*[8, 8]	14,048	17,952	75,744	39,840	1629	2450	517	6764	1139	17,952
*F*[9, 9]	17,784	22,734	95,904	50,436	2065	3098	654	8564	1441	22,734
*F*[10, 10]	21,960	28,080	118,440	62,280	2547	4000	807	10,576	1779	28,080

**Figure 3:**
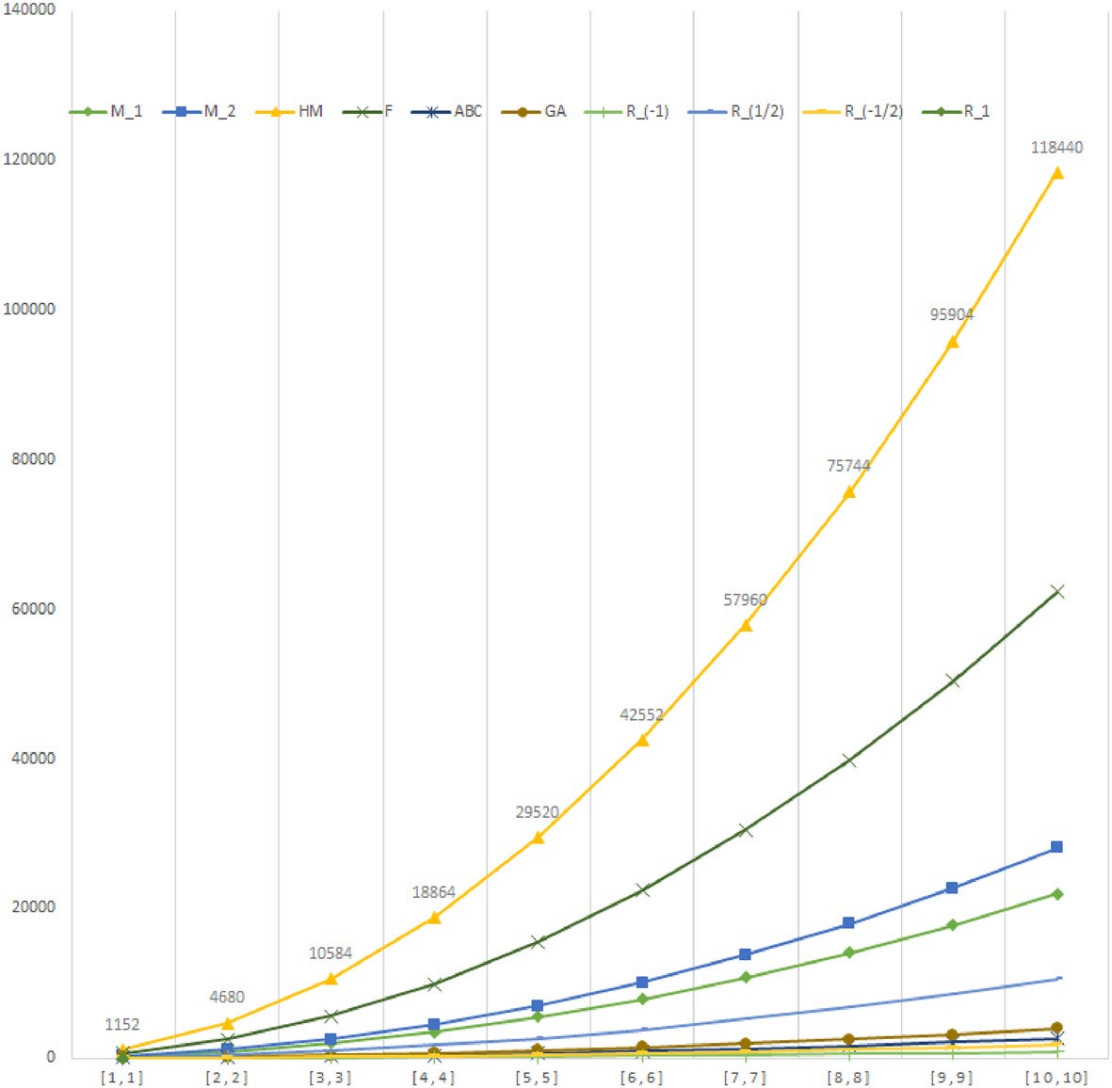
Comparison of indices on F[m,n], for m = n.

## Conclusion

In this paper, we determined the topological indices of supramolecular graph of Fuchsine acid, that would be helpful in computational chemistry. We have computed ABC index, geometric arithmetic index, general Randi$\acute{c}$ index, first and second Zagreb index, hyper zagreb index, harmonic index and forgotten index of supramolecule of fushine acid. The results of above mentioned indices are compared numerically as shown in Table 2, and graphically as shown in Fig. 3. Our computed results can be extend for the distance and resistance distance based topological indices of supramolecular structures.

## Data Availability

All data generated or analysed during this study are included in this published article.
